# An Epidemiological Prospective Study of Children’s Health and Annoyance Reactions to Aircraft Noise Exposure in South Africa

**DOI:** 10.3390/ijerph10072760

**Published:** 2013-07-03

**Authors:** Joseph Seabi

**Affiliations:** Department of Psychology, University of the Witwatersrand, Private Bag X3, Johannesburg 2050, South Africa; E-Mail: joseph.seabi@wits.ac.za; Tel.: +27-11-717-8331; Fax: +27-86-553-4926

**Keywords:** aircraft noise, annoyance, health, epidemiology, children, South Africa

## Abstract

The purpose of this study was to investigate health and annoyance reactions to change in chronic exposure to aircraft noise on a sample of South African children. It was the intention of this study to examine if effects of noise on health and annoyance can be demonstrated. If so, whether such effects persist over time, or whether such effects are reversible after the cessation of exposure to noise. A cohort of 732 children with a mean age of 11.1 (range = 8–14) participated at baseline measurements in Wave 1 (2009), and 649 (mean age = 12.3; range = 9–15) and 174 (mean age = 13.3; range = 10–16) children were reassessed in Wave 2 (2010) and Wave 3 (2011) after the relocation of the airport, respectively. The findings revealed that the children who were exposed to chronic aircraft noise continued to experience significantly higher annoyance than their counterparts in all the waves at school, and only in Wave 1 and Wave 2 at home. Aircraft noise exposure did not have adverse effects on the children’s self-reported health outcomes. Taken together, these findings suggest that chronic exposure to aircraft noise may have a lasting impact on children’s annoyance, but not on their subjective health rating. This is one of the first longitudinal studies of this nature in the African continent to make use of an opportunity resulting from the relocation of airport.

## 1. Introduction

Aircraft noise emissions appear to be annoying, largely because of their intermittent nature. A meta-analysis study revealed that among all transport noise sources, aircraft noise is considered the most annoying source [1]. Given that children are more susceptible to environmental stressors than adults because of reduced cognitive capacity to understand environmental issues and a lack of well-developed coping repertoires [2], it is crucial to understand how they perceive and react to changes in aircraft noise exposure. This is significant especially as a related study suggests that chronic exposure to aircraft noise may undermine children’s reading comprehension performance [3]. An understanding of the way environmental noise affects children’s development and functioning at home and school is fundamental to optimizing their learning potential and has implications for teaching practice and health.

Therefore, the present study investigates the health and annoyance reactions of children to change in aircraft noise exposure. Unlike previous studies that have explored the association between aircraft noise-exposure, annoyance and health, the strengths and uniqueness of the present study lies in its methodological design. Although laboratory studies that evaluate the impact of noise are crucial as they allow greater control of confounding variables (related to environmental conditions) than is possible in field studies, the participants are commonly exposed to only short bursts of noise during the experimental procedures and therefore generalization of the findings to chronically noise-exposed children is problematic [4]. Furthermore, the long-term exposure to aircraft noise on annoyance and health remains unknown due to most studies employing cross-sectional designs. Longitudinal studies that explore the associations between exposure to noise, annoyance, and health are required not only to provide understanding of causal pathways between these variables, but also to assist in the designing of preventive interventions. In the present study, subjective annoyance and health reactions of children in the high noise (HN) and low noise (LN) groups are investigated through longitudinal analyses. 

Although there is a growing body of literature conducted in Euro-Western countries [5,6,7,8,9] exploring noise annoyance, not much research appears to have been conducted on the African continent. To the best knowledge of the author, only three studies [10,11,12] conducted in this area could be located within the African continent and even so, with participants aged 12 years, and above. Furthermore, these studies adopted a cross-sectional research design, which has its own limitations. Noise pollution is often a forgotten environmental problem that is steadily growing in developing countries [13], where compliance with noise regulations is known to be weak [14]. South Africa as a developing country is no exception, since urban development, economic growth and the related growth in transportation, are the major pressures increasing the levels of noise. Therefore it is crucial to determine how a developing country such as South Africa fares in comparison to developed countries. 

### 1.1. Noise Annoyance and Health

Noise annoyance encompasses broad psychological feelings which include irritation, discomfort, distress, frustration, and offence (among others) when noise interrupts one’s psychological state or ongoing activities [15], and interferes with an individual’s quality of life. Noise could therefore indirectly result in poor health, whereby noise annoyance from chronic noise exposure may cause prolonged activation of physiological responses such as increased blood pressure, heart rate and endocrine outputs [16]. A cross-sectional study conducted near Schiphol and Heathrow airports demonstrated adverse effects of aircraft noise on blood pressure for 9–10 year old children [17], and similar findings were found elsewhere with children [18,19]. However inconsistent findings were demonstrated on psychological health. Children attending school within the vicinity of the Heathrow Airport were found to have higher levels of psychological distress and prevalence of hyperactivity [20]. In the Munich Airport Study, which adopted a prospective longitudinal design, effects of aircraft noise prior to and following the opening of the new airport as well as effects of chronic noise and its reduction at the old airport (*i.e.*, 6 and 18 month post relocation), were studied in 326 children aged 9 to 13 years [21]. On the basis of the three time points the children were investigated at the two airports, the findings demonstrated a significant decrease of total quality of life (*i.e.*, psychological, physical, social and functional daily life) 18 months after aircraft noise exposure as well as a motivational deficits operationalized by fewer attempts to solve insoluble puzzles in the new airport area. Quality of life became worse in children exposed to noise 18 months after the opening of the airport.

Conversely, in the largest epidemiological RANCH study, no effect of aircraft or road traffic noise was found on psychological distress [22]. Similar findings were found in 266 school children; thereby suggesting that exposure to chronic noise is not subjectively stressful [23]. Clark and Stansfeld concluded that noise exposure may not be associated with serious psychological illness though it may impact on the well-being and quality of life of children [16]. Given the lack of longitudinal research in this field, children’s subjective health reactions to long-term exposure to aircraft noise are thus explored in this prospective study.

Although extensive research on noise annoyance was carried out on adults there is a dearth of studies concerning children’s annoyance reactions to noise in school settings [24]. Consistent associations between exposure to aircraft noise and children’s annoyance have been demonstrated in cross-sectional and laboratory studies conducted within the vicinity of the international airports in developed countries [7,9,25,26,27,28]. A survey of over 2,000 primary school children aged 7 to 11 years in the UK exposed to different noise sources found that children were not only aware of the noise but were also annoyed by noise [29,30]. Although these studies shed some light about the impact that exposure to aircraft noise may have, the crucial questions remain unanswered regarding the long-term effects of aircraft noise exposure. Specifically, more current literature is required to reveal whether prolonged exposure to aircraft noise results in high levels of annoyance, and whether such effects remain constant or dissipate after the cessation of exposure to noise. If such effects disappear, after how long do they vanish? 

Few longitudinal studies have examined the effects of persistent exposure throughout children’s development. In the School Environment and Health Study, Haines and colleagues [20] conducted a longitudinal study around the Heathrow Airport with children aged 8 and 11. Amongst their findings, exposure to aircraft noise was related to high levels of noise annoyance, though the annoyance response remained constant over a year with no strong evidence of habituation. These findings contradicted the conclusions reached from the follow-up Los Angeles Study, whereby indications of habituation of physiological stress response were suggested [31]. It was therefore postulated that how children respond to coping with environmental stress influences reports of annoyance, more than physiological responses. In a retrospective longitudinal Munich Airport Study that took advantage of a naturally occurring experiment, which no other studies are yet to replicate, children’s affective responses to noise were investigated among 135 learners with a mean age of 10.78 [6]. Children living in noisier areas were significantly more annoyed by noise than those not exposed to noise. However, when the airport closed down, the annoyance diminished. Quite recently, Clark and her colleagues undertook a six-year follow-up of the UK RANCH cohort of children who were exposed to aircraft noise at primary and high schools around the Heathrow Airport [32]. These children significantly reported higher noise annoyance six years later at aircraft noise-exposed secondary school. No significant effects of noise on health outcomes were found. Although these findings demonstrate the impact of noise exposure on annoyance, they would have been more relevant for the present study had children who were tracked not been exposed to aircraft noise at high school, so that the longitudinal effects can be clearly demarcated. It would therefore be of interest to determine whether annoyance persisted or dissipated after the relocation of the airport.

### 1.2. Research Questions

The present study was undertaken to answer the following questions:

(1) Is there a statistically significant difference between children in the high noise (HN) and low noise (LN) groups on aircraft noise heard at *school* and *home* before and after relocation of the airport?
(2) Is there a statistically significant difference between HN and LN groups in the annoyance reaction from aircraft noise exposure at *school* and *home* before and after relocation of the airport?
(3) Is there a statistically significant difference between HN and LN groups on health scores before and after relocation of the airport?

## 2. Method

### 2.1. Context of the Study

The Durban International Airport was selected as a case study because it presented an opportunity to study the chronic effects of exposure to aircraft noise on learners’ health and annoyance reactions before and after it relocated to La Mercy, which is approximately 35 kilometers north of the city centre of Durban. According to the statistics provided by the Airports Company South Africa, this airport is the third busiest airport in South Africa, following OR Tambo International Airport in Johannesburg and Cape Town International Airport, and it is the ninth busiest airport in Africa [33]. 

The children in the present study came from five co-education public schools that were selected according to the noise exposure of the school area. Two highly exposed schools (HN group) were selected as the study population for the aircraft noise exposure area. The windows, walls, façade of the schools were not sound insulated. The low noise group comprised schools in locations not exposed to aircraft noise, but that matched the socio-demographic characteristics (such as age, language spoken at home, and social deprivation) of the high noise group. Schools located outside of the flight paths were selected by visual inspection of the airport. This study is conducted under the auspices of the Road Traffic and Aircraft Noise and Children’s Cognition and Health in South Africa (RANCH-SA). This project involves a cross-sectional, longitudinal design with children in Grades 5 through to Grade 8, who were attending schools in areas where there were high levels of aircraft noise. The aim of the project is to assess the effects of exposure to aircraft noise on the cognitive performance and health of primary school children. In the present paper, health and annoyance subjective reactions of children exposed to aircraft noise are investigated. This will ensure that when chronic effects of aircraft noise exposure on cognitive performance are investigated, factors that moderate or compound the associations are controlled.

### 2.2. Research Design

Given the difficulties of conducting long-term laboratory studies as they introduce ethical concerns around exposing participants to noise over period of time, which can potentially be harmful to their health, the current study adopts an epidemiological prospective field study design. This design is an “important method for identifying risk factors in epidemiological studies and it enables a stronger case for causation to be made because it is possible to demonstrate whether a proposed factor causes the development of the disease” (p.7 in [34]). This is the third longitudinal study to make use of a naturally occurring experiment resulting from the relocation of the airport and it involves within-group comparisons whereby measurements over three-time periods on the same children were made.

### 2.3. Participants

This paper is based on a cohort of 732 children with a mean age of 11.1 (range = 8–14) who participated at baseline measurements in Wave 1 (2009). A cohort of 649 (mean age = 12.3; range = 9–15) and 174 (mean age = 13.3; range = 10–16) children were reassessed after the relocation of the airport in Wave 2 (2010) and Wave 3 (2011), respectively.

**Table 1:** The socio-demographic characteristics of the high noise and low noise groups.

Socio-demographic characteristic	Low Noise	High Noise	OR	95% CI
**Wave 1**				
**Boys**	49%	51%	0.92	0.69–1.23
**English**	55%	59%	0.83	0.62–1.12
**Deprived**	30%	40%	0.62	0.46–0.85
**Wave 2**				
**Boys**	50%	50%	1	0.73–1.36
**English**	58%	62%	0.85	0.61–1.18
**Deprived**	31%	39%	0.70	0.50–0.99
**Wave 3**				
**Boys**	49%	54%	0.8	0.44–1.48
**English**	67%	53%	1.8	0.96–3.41
**Deprived**	43%	51%	0.73	0.39–1.35

There was a high attrition of participants in Wave 3 because permission to follow-up children in Grade 8 (*i.e.*, new schools) was not granted by some of the school principals, as well as the bad weather during the assessment day, which resulted in many children not coming to school. Research indicates that although prospective longitudinal studies are one of the strongest research methodologies for studying aetiological mechanisms [35], they are vulnerable to participant attrition [36]. Table 1 illustrates the socio-demographics of the sample.

### 2.4. Procedure

Written permission was obtained from the education authorities and from the parents to allow their children to participate in the study. The children were informed of the limits of confidentiality, as well as the voluntary nature of their participation. Informed assent from the children was thus obtained. On the day of testing, the assessment administrators introduced themselves according to the RANCH-SA script, which avoided the word ‘noise,’ so not to influence participants’ perceptions of the study, and the project was introduced as an environmental study. They were trained in advance on standard assessment protocol and how to administer the actual tests. The measurements were group-administered in the classrooms in the morning between 8 a.m. and 10 a.m. The pre-test measures were administered in Wave 1 before relocation of the airport and post-test measurements took place in Wave 2 and in Wave 3. Analyses presented in this paper are therefore of the 2009, 2010 and 2011 cohorts. Each testing procedure began with practice items to ensure that participants understood what was required in the assessment. Completed tests were placed in a coded envelop straight after the assessment was completed. The children were offered chips and juices for participation in the study.

### 2.5. Instruments

#### 2.5.1.Biographical Questionnaire

Information pertaining to participants’ gender, age, and languages was obtained from biographical questionnaires completed by the participants and parents. The child questionnaire was administered in print form and completed before the assessment. The parent questionnaire was sent beforehand to participants’ parents and collected from each child on the day of the experiment. Socio-economic status was assessed by the percentage of children eligible for free meals at school, since research indicates that there is a “significant correlation between the free school meal ratio and a range of census indicators representative of socio-economic status (p. 21 in [37]). A criterion for a child to be eligible for a free school meal is that the child’s caregiver should be receiving a government social grant.

#### 2.5.2. Noise Annoyance

Annoyance assessment measures regarding community response to aircraft noise often involve a participant rating his or her annoyance ratings on a numerical category scale; while in other studies participants are asked about noise interference with other activities [38]. In the present study, annoyance to aircraft noise was assessed with seven adapted questions [39], which measured the level of annoyance on a four-point Likert scale (never, sometimes, often, always) as experienced by the children when they heard aircraft and road traffic noise. The higher the score the higher the noise annoyance level (range 0–4).

#### 2.5.3. Child General Health

This questionnaire was adapted from the Child General Health questionnaire based on the largest epidemiological study to date on aircraft and road traffic noise [22]. Specifically, children were instructed to rate their health on a five-point rating scale (1 = very good to 5 = very bad). In addition they responded on a five-point rating scale (1 = never to 5 = every day) indicating whether they felt like vomiting, and experienced headache, tummy-ache, as well as difficulty sleeping (including waking up at night and feeling sleepy during daytime) in the last month.

#### 2.5.4. Perceived Noise Exposure

Children self-reported exposure to noise at school and home was measured from one source of environmental noise, aircraft. They were required to indicate if they heard noise and whether they were annoyed by such noise. They responded on a four-point rating scale (1 = never to 4 = always).

**Figure 1:**
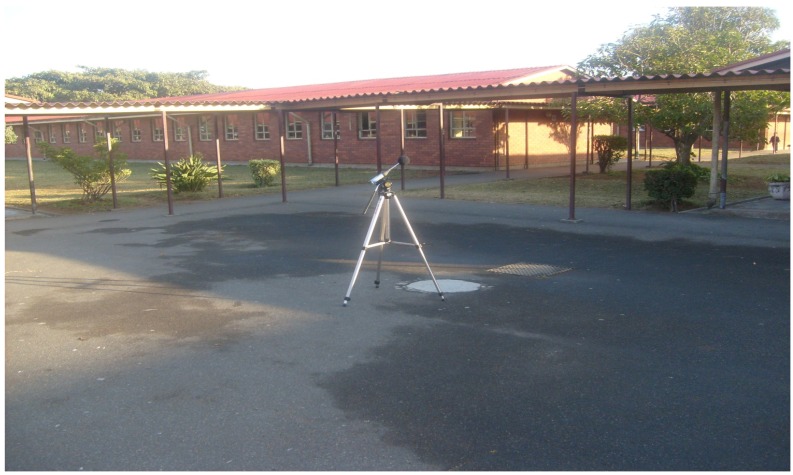
Sound level meter.

#### 2.5.5. Noise Measurements

**Figure 2:**
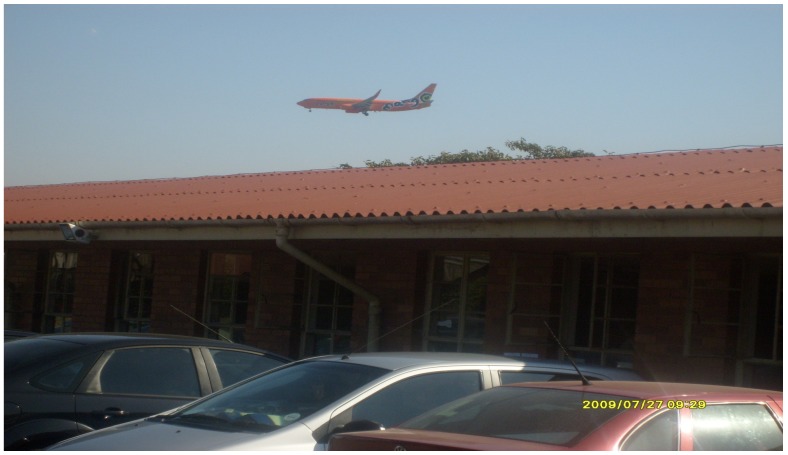
A sample of an aircraft flying over a school.

The instrument used to measure noise was a SVAN 955 Type 1 sound level meter (see Figure 1). A Rion NC74 acoustic calibrator was used to check the instrument calibration before and after the measurements were performed. Noise measurements were taken during the testing period (8 a.m. to 10 a.m.) outside the classrooms in order measure aircraft noise levels. The baseline Leq noise measurements for the High Noise groups at the noise exposed schools near the flight path (Wave 1) varied from 63.5 to 69.9 Leq. Maximum noise levels varied from 89.8 to 96.5dBA Lamax. In the case of the Low Noise groups at schools in relatively quieter areas, noise measurements during Wave 1 testing yielded results of 54.4 to 55.3 Leq and 73.2–74.3 Lamax. Noise measurements during Waves 2 and 3 when aircraft were gone produced results at the formerly noise exposed schools of 55.2 Leq and maximum noise levels of 60.8 to 71.2 Lamax. Levels at the quieter schools were averages of 50.5 to 57.9 Leq and 60.6 to 70.5. No measurements were conducted at the children’s homes due to limited resources but the schools were located within a walking distance. A sample of an aircraft flying over one of the noise-exposed schools in Wave 1 is presented in Figure 2.

### 2.6. Statistical Analysis

Statistical Analysis System (SAS) version 9.2 was utilised to conduct statistical analyses. In line with the previous study (p.470 in [40]) “all *F* tests with repeated measures of wave were treated as multivariate analyses of variance, MANOVAs, rather than univariate analyses of variance, ANOVAs. These MANOVAs yield higher *p* values and thus are more conservative, than the corresponding univariate epsilon-corrected Greenhouse-Geisser ANOVAs.” Effect estimates were presented as odds ratios (ORs) with 95% confidence intervals (CIs) for socio-demographic characteristics.

## 3. Results

### 3.1. Perception of Noise at School

As illustrated in Table 2, the HN group demonstrated significantly high mean scores in Wave 1 (*F*_1, 732_ = 104.29, *p* = 0.00) and Wave 2 (*F*_1, 649_ = 13.82, *p* = 0.00) on aircraft noise heard at school than the LN group. However, there was no significant difference (*F*_1, 174_ = 0.67, *p* = 0.41) between the two groups on aircraft noise in Wave 3. These results imply that children in the HN group perceived more aircraft noise at their school environment before and after the relocation of the airport than those in quieter environments (LN group).

**Table 2:** Perception of noise at school and home.

	Low Noise Mean	High Noise Mean	Difference Score (95% CI)	DF, N, *F*, *p*-value
**Wave 1**				
**School Item 1**	1.93	2.72	0.78 (−0.93–0.63)	(1, 732), F = 104.29, *p* = 0.00 *
**School Item 2**	1.83	2.88	0.05 (−1.20–0.91)	(1, 732), F = 213.96, *p* = 0.00
**Home Item 1**	2.43	2.38	0.05 (−0.09–0.19)	(1, 732), F = 0.49, *p* = 0.48
**Home Item 2**	2.08	2.38	−0.29 (−0.44–0.15)	(1, 732), F = 16.43, *p* = 0.00 *
**Wave 2**				
**School Item 1**	1.93	2.20	−0.26 (−0.40–0.12)	(1, 649), F = 13.82, *p* = 0.00 *
**School Item 2**	1.94	2.21	0.27 (−0.42–0.12)	(1, 649), F = 12.87, *p* = 0.00 *
**Home Item 1**	2.22	2.01	0.20 (0.07–0.34)	(1, 649), F = 9.04, *p* = 0.02 *
**Home Item 2**	1.96	2.37	−0.40 (−0.56–0.24)	(1, 649), F = 24.18, *p* = 0.00 *
**Wave 3**				
**School Item 1**	1.71	1.63	−0.08 (−0.11–0.27)	(1, 174), F = 0.67, *p* = 0.41
**School Item 2**	1.42	1.63	−0.21 (−0.41–0.00)	(1, 174), F = 4.06, *p* = 0.04 *
**Home Item 1**	1.95	1.74	0.20 (−0.00–0.42)	(1, 174), F = 3.62, *p* = 0.05 *
**Home Item 2**	1.71	1.63	0.08 (−0.16–0.32)	(1, 174), F = 0.43, *p* = 0.50

Notes: 1 = Hear aircraft noise; 2 = Annoyance from aircraft noise; * *p* < 0.05

**Figure 3:**
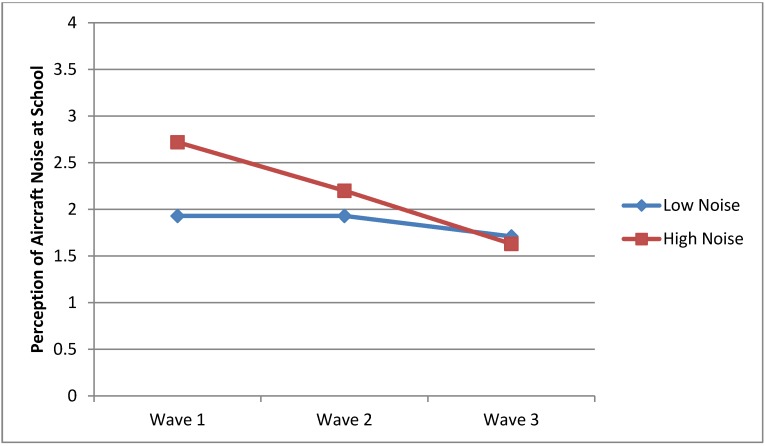
Perception of aircraft noise at school.

The present study was also interested in the potential interaction between Wave and Group on aircraft noise perceived at school. As illustrated in Figure 3, there were significant interactions (*F_2_*_, 174_ = 3.93, *p* = 0.02) similar to trends of the main effects, where the mean scores of the HN group were substantially higher than that of the LN group on aircraft traffic noise in Wave 1 and Wave 2, but not in Wave 3. 

### 3.2. Perception of Noise at Home

**Figure 4:**
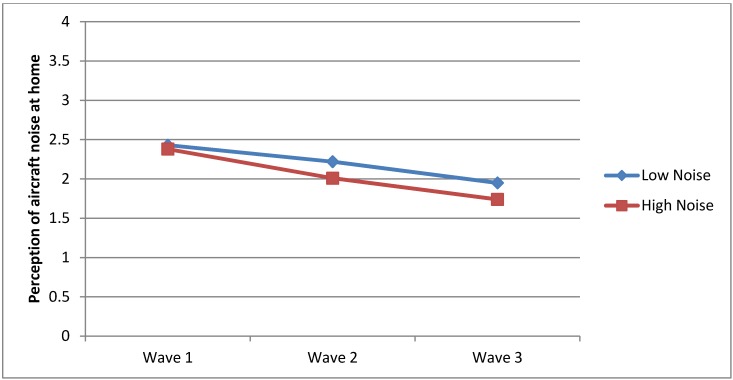
Perception of aircraft noise at home.

Question One also investigated whether there was a significant difference between the HN and LN groups on aircraft noise heard at home before and after relocation of the airport. As shown in Table 2, there was no significant difference between the groups in Wave 1 (*F*_1, 732_ = 0.49, *p* = 0.48). However, the mean scores of children within the LN group were substantially greater than that of the HN group in Wave 2 (*F*_1, 649_ = 9.04, *p* = 0.02) and Wave 3 (*F*_1, 174_ = 3.62, *p* = 0.05). These findings were not expected especially given that the LN group was not exposed to aircraft noise. Figure 4 presents graphic visual presentation of these results.

### 3.3. Annoyance Reactions at School

**Figure 5:**
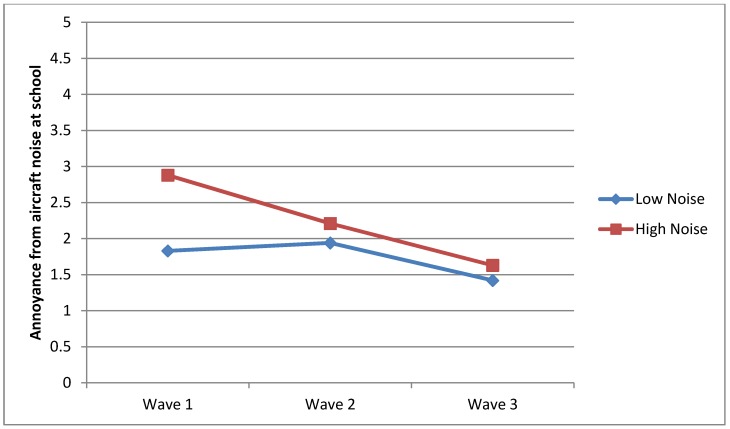
Annoyance reactions from aircraft noise at school.

In order to elucidate the impact of noise perceived at school, Question Two examined whether there was a significant difference in the annoyance reaction between the HN and LN groups throughout all the waves as a function of aircraft noise exposure. The HN group demonstrated statistically significant high mean scores than the LN group in Wave 1 (*F*_1, 732_ = 213.96, *p* = 0.00), Wave 2 (*F*_1, 649_ = 12.87, *p* = 0.00) and Wave 3 (*F*_1, 174_ = 4.06, *p* = 0.04), as illustrated in Table 2. Visual presentation of Figure 5 indicates that while children within the HN group were substantially annoyed by aircraft noise before it was decommissioned, the effects narrowed despite the significance effects remaining.

**Figure 6:**
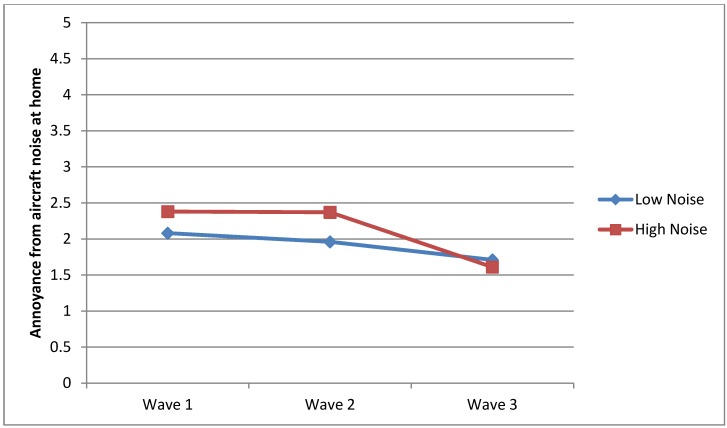
Annoyance reactions from aircraft noise at home.

### 3.4. Annoyance Reactions at Home

Question Two also investigated whether there was a significant difference between the HN and LN groups in the annoyance reaction from aircraft noise exposure at home throughout all the waves. As presented in Table 2, the HN group demonstrated significantly greater scores than the LN group on annoyance reactions from aircraft noise in Wave 1 (*F*_1, 732_ = 16.43, *p* = 0.00) and in Wave 2 (*F*_1, 649_ = 24.18, *p* = 0.00). No significant effects were observed in Wave 3 (*F*_1, 174_ = 0.43, *p* = 0.50). The presence of interaction effects are illustrated in Figure 6.

**Table 3:** Child Self-Reported Health.

	Low Noise Mean	High Noise Mean	Difference Score (95% CI)	DF, N, *F*, *p*-value
**Wave 1**				
**Item 1**	1.90	1.75	0.17 (0.03–0.32)	(1, 732) F = 6.20 *p* = 0.01 *
**Item 2**	2.04	2.05	−0.01 (−015–0.13)	(1, 732) F = 0.02 *p* = 0.88
**Item 3**	2.00	2.00	−0.11 (−0.27–0.03)	(1, 732) F = 2.30 *p* = 0.12
**Item 4**	1.94	1.94	0.06 (−0.09–0.22)	(1, 732) F = 0.59 *p* = 0.44
**Item 5**	1.95	2.06	−0.11 (0.24–0.01)	(1, 732) F = 2.87 *p* = 0.09
**Wave 2**				
**Item 1**	1.77	1.74	−0.02 (−0.09–0.14)	(1, 649) F = 0.18 *p* = 0.66
**Item 2**	1.97	2.04	0.66 (−0.21–0.88)	(1, 649) F = 0.76 *p* = 0.38
**Item 3**	1.86	1.91	−0.05 (−0.20–0.10)	(1, 649) F = 0.42 *p* = 0.51
**Item 4**	1.74	1.89	−0.15 (−0.31–0.01)	(1, 649) F = 3.27 *p* = 0.07
**Item 5**	1.85	1.93	−0.07 (−0.20–0.04)	(1, 649)F = 1.50 *p* = 0.22
**Wave 3**				
**Item 1**	1.73	1.77	−0.04 (−0.25–0.16)	(1, 174) F = 0.17 *p* = 0.67
**Item 2**	1.96	1.97	−0.01 (−0.28–0.25)	(1, 174) F = 0.01 *p* = 0.91
**Item 3**	1.75	1.82	−0.07 (−0.35–0.20)	(1, 174) F = 0.27 *p* = 0.60
**Item 4**	1.86	1.75	0.11 (−0.19–0.41)	(1, 174) F = 0.51 *p* = 0.47
**Item 5**	1.95	2.09	−0.14 (−0.42–0.13)	(1, 74) F = 1.05 *p* = 0.30

Notes: 1 = General health; 2 = Headache; 3 = Vomit; 4 = Tummy-ache; 5 = Difficulty Sleeping; * *p* < 0.05

### 3.5. Child Self-Reported Health

Question Three compared children’s self-reported health scores between the HN and the LN groups throughout all the waves. The LN group demonstrated significantly poorer health score than the HN group in Wave 1 (*F*_1, 732_ = 6.20, *p* = 0.01), as illustrated in Table 3. However, there was no significant difference between the two groups in Wave 2 (*F*_1, 649_ = 0.18, *p* = 0.66) and Wave 3 (*F*_1, 174_ = 0.17, *p* = 0.67). There were also no statistically significant differences observed between the HN and the LN groups with regards to headache, vomiting, tummy-ache, and difficulty sleeping in all the waves.

## 4. Discussion

This prospective study explored children’s perceived health and annoyance reactions to change in exposure to aircraft noise in South Africa. This is the first largest epidemiological prospective study in the African continent to explore the influence of chronic exposure to aircraft noise on children’s health and annoyance reactions. There were four main findings in the present study. First, children within the HN group continued to perceive substantial amount of noise despite the relocation of the airport than those in the LN group at school. Second, although there was no significant difference in the perception of noise between the groups at Wave 1 at home, learners in the LN group perceived greater noise levels than their counterparts at Wave 2 and Wave 3. Third, learners within the HN group experienced high levels of annoyance throughout all the waves at school and home (Wave 1 and Wave 2). Fourth, despite the LN group exhibiting poor health scores at Wave 1, there was no significant difference between the groups on health outcomes in Wave 2 and Wave 3. Taken together, these findings suggest that chronic exposure to aircraft noise may have a lasting impact on children’s annoyance, but not on their subjective health rating.

### 4.1. Perception of Noise

Children exposed to high levels of aircraft noise (HN) perceived substantial amount of noise prior to and following the relocation of the airport than those in the LN group at school. These findings corroborate previous research which found that forty-two percent of children heard aircraft noise at home [41]. Although it was expectable for the HN learners in the present study to experience high levels of aircraft noise given that their schools were located under flights path, it is of significant interest to note that they continued to perceive substantial amount of noise at school despite the relocation of the airport to another area. It seems that these children were accustomed to noise exposure. Indeed, Evans and Lepore report that children may adapt to the distracting chronic noise by filtering or tuning out both unwanted auditory stimuli and relevant auditory stimuli [42]. Although children may find this cognitive strategy helpful, it is cautioned that the tendency of children to tune out noise may become over-generalised in such a way that they tune out stimuli indiscriminately [22]. This tuning out cognitive strategy may lead children exposed to noise to have poorer ability to sustain attention in the classroom, which may affect concentration and learning over time, even in the absence of noise exposure [20]. It was also found in a different study that children who used this cognitive strategy (tuning out) had deficits in attention, auditory discrimination and speech perception [43]. The findings from the present study have implication to education, especially because children spend much of their time listening in classroom. Successful communication does not depend only on the skill of the educator to impart knowledge, but also on whether the educator can be heard correctly by the children [44]. It is therefore argued that poor listening environments have unfavourable effects on children’s ability to attend and process relevant aspects of the acoustical signals in classrooms and compromise learning and performance [45]. It thus seems imperative that the acoustical conditions in classrooms should foster children’s listening.

The findings from the present study also revealed no significant difference between the HN and LN groups on noise heard at home at Wave 1. However, children in the LN group perceived greater noise levels at home than their counterparts at Wave 2 and Wave 3. This finding was not expected and it seems that other sources of noise may have possibly emerged given that this group was not located within the vicinity of the airport. By adopting a quantitative approach that involved administration of questionnaires, it appears that the crucial information about other sources of information may have been missed out. Burns cautions that exclusive reliance on quantitative approaches can become an end in itself given that participants are restricted to options predefined by the researcher [46].

### 4.2. Aircraft Noise and Annoyance

The findings of the present study revealed higher annoyance among children within the HN group in all the waves at school and in Wave 1 and Wave 2 at home. These results are consistent with empirical research, which has shown that children exposed to noise are annoyed by exposure to aircraft noise [8,9,32,47]. Different from the previous studies though, is that children in the HN group remained annoyed by noise even after the relocation of the airport. These results are inconsistent with the longitudinal study conducted at the Munich Airport, which found that children living in noisier areas were significantly more annoyed by noise but when the airport closed down, the annoyance diminished [6]. The results of the present study seems to suggest that chronic exposure to aircraft noise may have a lasting impact on children’s development and therefore children should be protected from such environmental hazards. However, since the HN group continued to perceive noise after the relocation of the airport, and they remained annoyed, could these findings be attributed to other sources of noise not measured? This warrants further investigation.

It is surprising that children within the HN group were also highly annoyed by exposure to noise at home at Wave 1 and Wave 2, especially because they perceived less noise at home in comparison to their counterparts (as per the previous section). Given that LN children perceived substantial amount of noise at Wave 2 and Wave 3, it would have been expectable for this group to be highly annoyed at home. These results may point toward a stress related effect. Children in the HN group were exposed to higher amount of stress at school and this sympathetic overstimulation may have been transferred to the children’s home although the stress did not persist.

### 4.3. Aircraft Noise and Health

The results showed that the general health of the LN children was poor at Wave 1. This result is unfathomable especially because efforts were made during the conceptualization and piloting phase to ensure that children in the HN and LN were from similar socio-economic and health backgrounds. It is further surprising that their health was relatively poor only at Wave 1 and yet they were not exposed to aircraft noise. It seems that other factors (e.g., air pollution, noise pollution from road traffic, construction, and so on) beyond the scope of this study may have been responsible for these results.

There was no significant difference between the HN and LN children on the other health-related outcomes (e.g., headache, vomit, tummy-ache, and difficulty sleeping) in all the waves. Consistent with these results are the findings from one of the largest epidemiological RANCH study, which established no effect of aircraft or road traffic noise on health [22], even six years later [32]. In a qualitative study, Haines and her colleagues reported that children did not perceive noise pollution to have adverse effects on their health [26]. It thus seems that exposure to aircraft noise does not have an adverse effect on children’s self-reported health outcomes. However, these findings contradict those found in the Munich Airport Study, which demonstrated a significant decrease of total quality of life up to 18 months after relocation of the airport [21]. It is therefore recommended that future studies should either use the same instrument that measured the total quality of life in the Munich Study or objective measures of health to determine whether or not aircraft noise exposure impacts negatively on health.

### 4.4. Implications of the Findings

Although there are numerous economic and social welfare benefits of air transportation (*i.e.*, commercial aircraft, jet, aviation aircraft and helicopter), they also come at a cost. It is evident from the results of the current epidemiological study that exposure to aircraft noise results in substantial levels of annoyance. Given that children who were exposed to aircraft noise continued to experience greater annoyance, following the relocation of the airport, chronic aircraft noise-exposure seems to have a lasting impact on children’s functioning. These effects appear not to be reversible. It is therefore crucial that policy makers and airport officials ensure that children’s school environments are conducive to their learning and development, that environmental hazards such as noise pollution are avoided and/or eliminated. Aircraft noise exposure does not have adverse effects on health-related outcomes and this could possibly be as a result of the subjective measures that were used to assess health in this study. Future studies should make use of objective measurements.

### 4.5. Strengths and Limitations

To best knowledge of the author, this longitudinal field study is the first largest study to date to examine the effects of aircraft noise exposure on children’s health and annoyance reactions within the African continent. A major limitation of the study is that, while the analyses are based on a longitudinal data (2009–2011), the 2011 cohort was very small because significant proportions of the participants were lost due to attrition. Noise measurements were only carried out in schools and not in the children’s homes due to limited resources. Another limitation relates to the exclusive focus on aircraft noise and not on the other sources of noise (such as road traffic, construction, railway noise *etc*.), which may have compounded the results. Future studies should use mixed-methods design to avoid restricting or limiting participants’ responses.

## 5. Conclusions

The overall goal of the present study was to investigate the long-term effects of chronic exposure to aircraft noise on health and annoyance reactions on a sample of South African children. It was the intention of this study to examine if there are effects of noise on health and annoyance. And if so, whether such effects persist over time, or whether such effects are reversible after the cessation of exposure to aircraft noise. The findings revealed that despite the relocation of the airport, the children who were exposed to chronic aircraft noise continued to be substantially annoyed than children from quieter environment. Aircraft noise exposure did not have adverse effects on children’s health.
